# Young Adults with Autism Spectrum Disorder Show Early Atypical Neural Activity during Emotional Face Processing

**DOI:** 10.3389/fnhum.2018.00057

**Published:** 2018-02-22

**Authors:** Rachel C. Leung, Elizabeth W. Pang, Evdokia Anagnostou, Margot J. Taylor

**Affiliations:** ^1^Diagnostic Imaging, Hospital for Sick Children, Toronto, ON, Canada; ^2^Neurosciences and Mental Health Program, Research Institute, Hospital for Sick Children, Toronto, ON, Canada; ^3^Department of Psychology, University of Toronto, Toronto, ON, Canada; ^4^Division of Neurology, Hospital for Sick Children, Toronto, ON, Canada; ^5^Department of Paediatrics, University of Toronto, Toronto, ON, Canada; ^6^Bloorview Research Institute, Holland-Bloorview Kids Rehabilitation Hospital, Toronto, ON, Canada

**Keywords:** adults, autism spectrum disorder, emotional face processing, magnetoencephalography, social cognition

## Abstract

Social cognition is impaired in autism spectrum disorder (ASD). The ability to perceive and interpret affect is integral to successful social functioning and has an extended developmental course. However, the neural mechanisms underlying emotional face processing in ASD are unclear. Using magnetoencephalography (MEG), the present study explored neural activation during implicit emotional face processing in young adults with and without ASD. Twenty-six young adults with ASD and 26 healthy controls were recruited. Participants indicated the location of a scrambled pattern (target) that was presented alongside a happy or angry face. Emotion-related activation sources for each emotion were estimated using the Empirical Bayes Beamformer (*p*_corr_ ≤ 0.001) in Statistical Parametric Mapping 12 (SPM12). Emotional faces elicited elevated fusiform, amygdala and anterior insula and reduced anterior cingulate cortex (ACC) activity in adults with ASD relative to controls. Within group comparisons revealed that angry vs. happy faces elicited distinct neural activity in typically developing adults; there was no distinction in young adults with ASD. Our data suggest difficulties in affect processing in ASD reflect atypical recruitment of traditional emotional processing areas. These early differences may contribute to difficulties in deriving social reward from faces, ascribing salience to faces, and an immature threat processing system, which collectively could result in deficits in emotional face processing.

## Introduction

Autism spectrum disorder (ASD) is a neurodevelopmental disorder in which impairment in social functioning is a hallmark feature. Successful social functioning relies on not only verbal expression but also on the conveying of nonverbal cues, with one particularly important skill being the perception and interpretation of emotional facial expressions. Given that abilities for successful social interaction and facial emotion processing tend to be concomitant, it would be expected that individuals with ASD would have an impairment in facial emotion processing.

Findings however, have been inconsistent with some studies reporting intact emotional face processing and others reporting significant impairment. Incongruences in the literature have been suggested to reflect the sample demographics (e.g., age), task demands (e.g., stimulus exposure duration) and the dependent variables evaluated in the specific studies (Harms et al., 2010). Furthermore, use of compensatory mechanisms such as cognitive resources or linguistic strategies may also play a role in determining whether or not emotional face processing deficits are observed. Relative to typically developing peers, however, individuals with ASD have been shown to lack interest in the human face (Osterling and Dawson, 1994). Across normal development, individuals develop an expertise in face processing and perception of the intent of emotional expressions occurs automatically (Whalen et al., 1998). The notion that a network of cortical regions becomes functionally specialized, or selective, to processing certain “preferred” information, such as faces, with development, is in line with the Johnson’s seminal interactive specialization model (Johnson, 2000, 2011).

Thus, while individuals with ASD may make use of compensatory mechanisms to mask difficulties in affect processing, the process still lacks the automaticity that typically developing individuals have being “face experts”. Furthermore, while happy faces are socially rewarding for typically developing individuals, those with ASD do not appear to derive the same reward from positive faces (Sepeta et al., 2012). A lack of interest in human faces, compounded by a lack of social significance and reward of faces, exacerbates the poorer development of face expertise in ASD, which in turn has major implications for the perception and processing of emotional information conveyed by facial expressions.

An understanding of the biological mechanisms that support emotional face processing in ASD is crucial to understanding this deficit. A number of neural structures are recruited during affective processing. A seminal review by Haxby et al. (2002) described the role of these core emotional regions, identifying both cortical, including the anterior cingulate cortex (ACC) and subcortical regions, such as the amygdalae, as well as “extended” emotional regions including the anterior insula and prefrontal cortex. However, Pessoa (2008) also cautioned against a model of functional specialization of specific neuroanatomical structures and instead argued for a network approach in that affective processing recruits an interactive and dynamic system of neural regions that are also implicated in other functions.

Prior studies report atypical neural activity during emotional face processing in individuals with ASD (for a review see Harms et al., 2010). Functional magnetic resonance imaging (fMRI) studies have shown atypical amygdala and fusiform activation in individuals with ASD during emotional face processing, relative to typically developing individuals (e.g., Critchley et al., 2000; Ashwin et al., 2007; Deeley et al., 2007). Electrophysiological studies have similarly shown slowed or reduced neural responses during face processing in adolescents and adults with ASD (McPartland et al., 2004; O’Connor et al., 2005, but see Webb et al., 2010) or neural responses that failed to differentiate between specific emotions (Wagner et al., 2013). Further, while differences in early visual processing between youth with and without ASD to emotional cues of fear and anger were noted, youth with ASD did not show the early distinctive neural responses to fear vs. anger that was observed in typically developing youth (Malaia et al., 2017).

However, MRI or electroencephalography afford either good spatial or good temporal resolution, respectively, but not both. Being able to investigate both temporal and spatial properties of emotional face processing is important given the interactive network model and the rapidity of face processing. For example, early emotion-sensitive components at 100 ms can be seen both frontally and occipitally; however, while occipital sensitivity shows increased activation to fearful faces regardless of attentional demands, early frontal sensitivity is specific in response to implicitly processed emotional faces (Batty and Taylor, 2003; Bayle and Taylor, 2010). Thus, use of a neuroimaging modality such as magnetoencephalography (MEG) that yields both high spatial and temporal resolution, resolving neural activity on a millisecond and 5-mm scale (Hari et al., 2010; Hari and Salmelin, 2012), is ideal to study emotional face processing.

Despite the advantages of MEG, there have been a limited number of MEG investigations looking at face and affective processing in ASD. Reduced activity in right anterior temporal regions during face processing was reported as early as 30–60 ms in adults with ASD relative to controls (Bailey et al., 2005). In children with ASD, face and non-face stimuli evoked comparable activity, whereas in typically developing children, the 100 ms response to faces was significantly different than that of non-face stimuli; these results suggest that children with ASD may process non-face objects at a higher (extrastriate) level than faces (Kylliäinen et al., 2006). In a more recent study by Wright et al. (2012) in children with ASD, decreases in gamma power for anger, disgust and sadness in the left supramarginal and left precentral gyrus were noted whereas controls showed emotion-specific increases in gamma power. The authors suggested that gamma disruption may be a mechanism for difficulties in facial affect processing in ASD (Wright et al., 2012). Atypical insula, anterior and posterior cingulate and temporal and orbitofrontal activity during emotional face processing has also been shown in adolescents with ASD, which may underlie deficits in face processing and comprehension of social reward and punishment (Leung et al., 2015).

Many of the classic studies on emotional face processing have focussed on the expression of fear (e.g., Morris et al., 1998). While both anger and fear expressions are indicative of threat, Marsh et al. (2005) noted that the former facilitates avoidance-related behaviors while the latter, counter-intuitively, elicits approach-related behaviors. The difference between these two negative emotions lies in their social function: anger expressions represent a source of threat and facilitate aversive behavior while an expression of fear indicates submission, which plays a role in appeasement and facilitates affiliative behavior and social bonds (Marsh et al., 2005). Anger is also a more commonly encountered emotion, and its investigation may have greater ecological validity, given displays of anger occur in response to violation of social norms. Atypical processing of angry emotional stimuli is seen in those with ASD (Ashwin et al., 2007) and changes in anger processing with age have been reported (Lindner and Rosén, 2006). To date, there have been no investigations using angry faces in young adults with ASD to establish the spatio-temporal neural correlates of processing these salient stimuli. The present study determined the neural mechanisms underlying implicit emotional face processing in young adults with ASD using MEG and Empirical Bayesian Beamformer (EBB; Friston et al., 2006; Mattout et al., 2006) to localize sources of neural activity. We hypothesized reduced and delayed brain activation in those with ASD relative to typical adults, consistent with the reported difficulties with their processing of emotional faces.

## Materials and Methods

### Participants

The study included 52 young adults aged 19–36 years of age; 26 with ASD (8 females, 26.3 ± 4.2 years, age range = 19.2–36.3 years, IQ = 114.0 ± 16.8) and 26 control adults (8 females, 26.3 ± 4.1 years, age range = 19.7–36.5 years, IQ = 114.0 ± 9.4). Recruitment occurred via flyers posted around the Hospital for Sick Children and word-of-mouth. The Autism Diagnostic Observation Schedule, administered to the clinical group only (ADOS-G, ADOS-2; Lord et al., 2000, 2012; Rutter et al., 2012) and expert clinical judgment confirmed an ASD diagnosis in the clinical sample. Participant exclusion criteria included a history of neurological or neurodevelopment disorders (other than ASD in the clinical sample), use of psychotropic medications (for the control sample), acquired brain injury, IQ ± 70 and standard contraindications to MEG and MRI. Psychotropic medications that participants with ASD reported being on at the time of the study included Adderall, Celexa, Cipralex, Wellbutrin, Concerta, Cymbalata, Labox, Risperidone, Prozac, Imovane, Ativan, Sertraline, Zoloft and Abilify. This study was carried out in accordance with the recommendations of The Hospital of Sick Children Research Ethics Board with written informed consent from all subjects and in accordance with the Declaration of Helsinki. The protocol was approved by the The Hospital of Sick Children Research Ethics Board.

### Emotional Face MEG Task and Procedure

Emotional (happy or angry) or neutral faces were presented concurrently with a scrambled pattern (the scrambled image of the face, corrected for luminosity and color), on either side of a central fixation cross. Seventy-five face stimuli (25 of each facial expression: happy, angry, neutral; 13 male faces, 12 female faces) were selected from the NimSet Set of Facial Expressions (threshold: 80% minimum accuracy; Tottenham et al., 2009). To create the unique scrambled patterns for each face, face stimuli were modified by Adobe Photoshop. Each face stimulus was randomly divided into 64 cells, which were then mosaicked (15 cells per square), Gaussian blurred (10.0 degrees) and luminosity- and color-matched to the original images. The task consisted of 300 trials (50 trials of each expression in each hemifield); each face was presented twice in each hemifield. *Presentation* software^1^ was used for stimulus presentation, recording response latencies and accuracy. To minimize saccades, stimuli were presented for only 80 ms with a jittered inter-stimulus interval that varied from 1300 ms to 1500 ms. Participants lay supine in the MEG; task stimuli were back-projected onto a screen 79 cm in front of participants. Images subtended 6.9° and were presented parafoveally. Participants were asked to fixate on the central cross and indicate the location (left or right) of the target (scrambled pattern) using a response button box. All participants completed a set of practice trials prior to the neuroimaging session to ensure task comprehension.

### MEG Data Acquisition

A 151-channel CTF MEG system (Coquitlam, BC, Canada) was used to acquire MEG data at a 600 Hz sampling rate, third order spatial gradient and a recording bandpass of 0–150 Hz in a magnetically shielded room at the Hospital for Sick Children. Three fiducial coils placed on the left and right- pre-auricular points and the nasion were used to monitor head position and motion within the MEG dewar. Prior to completing the MRI, the fiducial coils were replaced with radio-opaque markers for MRI co-registration.

### MRI Data Acquisition

A T1-weighted MR image (3D SAG MPRAGE: PAT, TR/TE = 2300/2.96 ms, GRAPPA = 2, FA = 9°, FOV 25.6 × 25.6 cm, 240 × 256 matrix, 192 slices, slice thickness of 1 mm isotropic voxels) was acquired for each participant on a 3T MR scanner (MAGNETOM Tim Trio, Siemens AG, Erlangen, Germany) with a 12-channel head coil, as an anatomical underlay for each subject’s MEG, to ensure accurate source localization.

### Neuropsychological and Autistic Symptomology Measures

The Module 4 of the ADOS-G and ADOS-2 (Lord et al., 2000; Rutter et al., 2012), suitable for adults with fluent speech, is a semi-structured clinical assessment of autistic symptomology. Module 4 was administered to the clinical group. The Wechsler Abbreviated Scale of Intelligence (WASI; Wechsler, 2002) two-subtest version (vocabulary and matrix reasoning) was used to obtain an estimate of IQ in all participants.

### Preprocessing Steps

Statistical Parametric Mapping 12 (SPM12; Wellcome Trust Centre of Neuroimaging, London)^2^ implemented in MATLAB R2014b (The Mathworks, Natick, MA, USA) was used to pre-process and analyze MEG data. Data were first filtered with a fifth-order Butterworth filter with a bandpass at 1–50 Hz. Baseline-corrected epochs of 800 ms (from 200 ms pre-stimulus onset to 600 ms post-stimulus onset) were extracted. Epochs containing inter- and intra-trial movement in excess of 10 mm and 5 mm, respectively, were rejected. Independent Component Analysis (ICA; EEGlab)^3^ was used to identify and remove ocular and muscular artifacts on a trial-by-trial basis for each participant and condition. For each participant, a maximum of 30 components were examined and artifacts were removed based on visual analysis of the component. Further artifacts detection was performed in SPM by channel thresholding (bad channel threshold = 0.2, thresholded 2000 channels, excision window of 600 ms). Finally, trials were averaged within each emotion and collapsed across hemifields.

### Source Reconstruction and Statistical Analyses

The EBB (Friston et al., 2006; Mattout et al., 2006) was applied to localize sources of MEG activity using sliding time windows of 50 ms from 50 ms to 500 ms, overlapping by 25 ms (e.g., 100–150 ms, 125–175 ms). The SPM12 cortical mesh template in Montreal Neurological Institute (MNI) standard space was co-registered to MEG sensor space using the fiducial markers from each participant’s T1 MR image. A single shell head model was used to forward compute the gain matrix of the lead field model (Nolte, 2003). Beamformed images were spatially smoothed using a full-width half maximum Gaussian smoothing kernel of 12 mm.

2 (group: ASD, controls) × 2 (emotion: happy, angry) ANOVAs were conducted on beamformer images to determine areas of peak differences of main effects and interactions in neural activity for happy and angry faces. Given *a priori* hypotheses, *t*-tests were used to compare differences in functional brain activations between groups within each emotion (e.g., ASD vs. controls to angry faces) and within group (e.g., happy vs. angry in controls). Data were corrected for multiple comparisons by applying a Bonferroni error correction (*p*_corr_ < 0.05) to the results, which corrected the significance level for the number of spatial degrees of freedom (*df* = 30.15) in beamformer reconstruction (Wens et al., 2015; Urbain et al., 2017); only *p*-values less than 0.00166 were considered significant.

In voxels where there were significant between-group differences in neural activity (*p*_corr_ ≤ 0.001), source time courses were computed to estimate differences across participants, using a ranked sum test to identify significant (*p*_corr_ ≤ 0.001) differences in neural activation across time. All effects reported passed this significance level. Lastly, MRIcron software (Rorden, 2012) was used to create 3D renderings of significant differences in neural activity on spatially normalized brain images.

## Results

### Behavioral Findings

SPSS 24 software (SPSS Inc., Chicago, IL, USA) was used to analyze group effects in IQ, response accuracy and response latencies across emotions (Table 1). Repeated measures ANOVAs were conducted to examine group (ASD vs. control) and emotion (angry vs. happy) effects. There were no between-group differences in IQ (*t*_(36.68)_ = 0.00, *p* = 1.00). A 2 (emotion: happy, angry) × 2 (group: ASD, controls) repeated measures ANOVA showed no between-group effect on accuracy (*F*_(2,48)_ = 2.19, *p* = 0.124) or response latency (*F*_(2,48)_ = 0.194, *p* = 0.824).

**Table 1:** Summary of group IQ and implicit emotional face task performance.

Group	*N*	IQ	Emotion			
			Happy		Angry	
			Accuracy	Response latency	Accuracy	Response latency
ASD	26	114.00 ± 16.77	96.36 ± 3.74	369 ± 67	92.23 ± 11.11	371 ± 67
Controls	26	114.00 ± 9.63	93.27 ± 10.28	376 ± 63	96.64 ± 4.38	379 ± 62

*Task accuracy (out of 100%) and response latencies (ms) in young adults with and without ASD are listed. There were no significant differences in accuracy or response latency between groups*.

### MEG Findings

#### Between Group Comparisons

##### Angry faces

**Figure 1:**
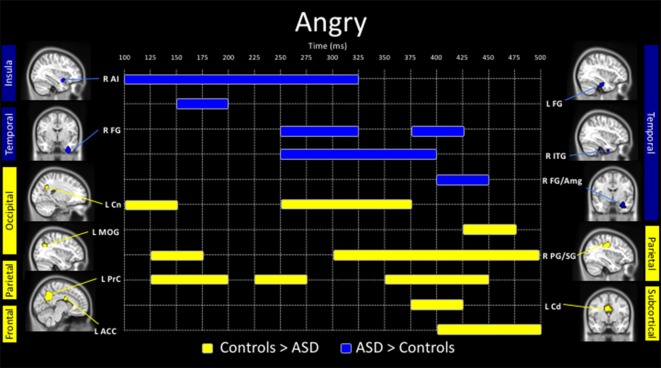
Spatiotemporal profile of significant between-group differences to angry faces (*p*_corr_ ≤ 0.001). Blue bars indicate regions and length of activation where young adults with autism spectrum disorder (ASD) showed significantly greater activation than control adults. Yellow bars show the length of activation of regions where adults with ASD showed reduced activation relative to controls. ACC, anterior cingulate cortex; AI, anterior insula; Amg, amygdala; Cd, caudate; Cn, cuneus; FG, fusiform gyrus; ITG, inferior temporal gyrus; MOG, middle occipital gyrus; PG/SG, postcentral/supramarginal gyrus; PrC, precuneus; L, left; R, right.

**Figure 2:**
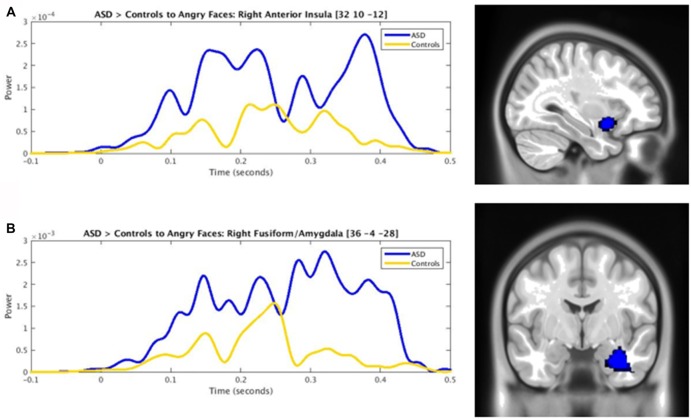
Time courses contrasting ASD (blue) and control (yellow) activity in the **(A)** right anterior insula and **(B)** right fusiform/amygdala to angry faces (*p*_corr_ ≤ 0.001).

The presentation of angry faces elicited greater activity in adults with ASD than controls (Figure 1) in the right inferior temporal (250–400 ms), left fusiform (150–200 ms), right fusiform (250–325 ms, 375–425 ms) and right amygdala/fusiform (400–450 ms). The right anterior insula also showed greater prolonged activity (100–325 ms) in adults with ASD compared to controls (Figure 1, blue bars). Time courses for both groups in the right anterior insula and right fusiform/amygdala were computed (Figures 2A,B).

In response to angry faces, adults with ASD also showed under-activation, relative to controls, in occipital areas, including the left cuneus (100–150 ms, 250–375 ms) and left middle occipital (425–475 ms) gyrus, and parietal areas, including the left precuneus (125–200 ms, 225–275 ms, 350–450 ms) and right postcentral/supramarginal (125–175 ms, 300–500 ms) gyrus (Figure 1, yellow bars). Adults with ASD also showed less activity in the left caudate (375–425 ms) and left ACC (400–500 ms).

##### Happy faces

**Figure 3:**
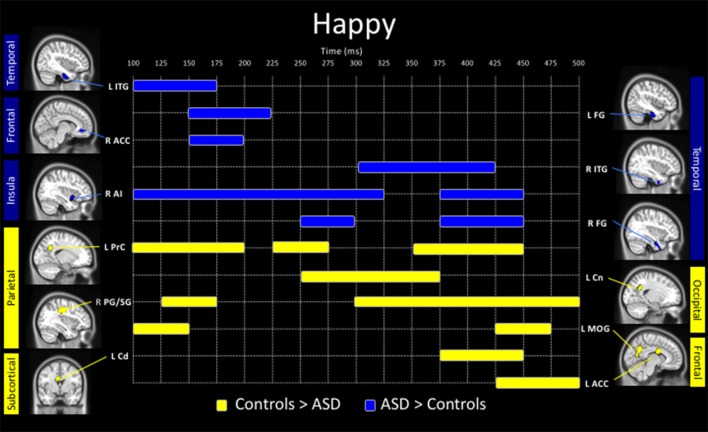
Spatiotemporal profile of significant between-group differences to happy faces (*p*_corr_ ≤ 0.001). Blue bars denote regions and length of activation significantly greater activity in young adults with ASD relative to controls. Yellow bars denote areas and length of activation of elevated activity in young adults with ASD relative to controls. ACC, anterior cingulate cortex; AI, anterior insula; Amg, amygdala; Cd, caudate; Cn, cuneus; FG, fusiform gyrus; ITG, inferior temporal gyrus; MOG, middle occipital gyrus; PG/SG, postcentral/supramarginal gyrus; PrC, precuneus; L, left; R, right.

Adults with ASD showed both increased and decreased activity, relative to controls, in a number of brain areas to the presentation of happy faces (Figure 3). In temporal areas, greater activity in the ASD group, was seen in the left inferior temporal gyrus (100–175 ms), left fusiform (150–225 ms), right ITG (300–425 ms) and right fusiform gyrus (250–300 ms, 375–450 ms). Those with ASD also showed greater right anterior cingulate (150–200 ms) and anterior insula activation (100–325 ms, 375–450 ms) all in Figure 3 (in blue).

Underactivity in the clinical group compared to controls, was noted in the left middle occipital gyrus (100–150 ms, 425–475 ms), the left precuneus (100–200 ms, 225–275 ms, 350–450 ms), left cuneus (250–375 ms), right postcentral/supramarginal (125–175 ms, 300–500 ms), left caudate (375–450 ms) and left ACC (425–500 ms; Figure 3, in yellow).

#### Within Group Comparisons

**Figure 4:**
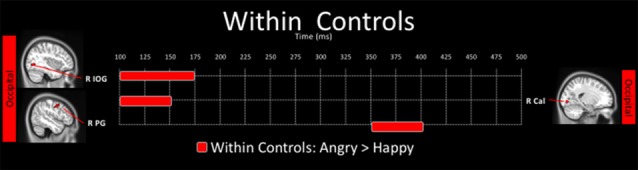
Spatiotemporal profile of significant within-group differences where activation to angry are significantly greater than to happy faces in control adults (*p*_corr_ ≤ 0.001). There were no significant differences in the angry < happy contrast in control adults, and there were no within-group differences for the ASD group.

There were no significant differences in neural activation between happy and angry faces in the young adults with ASD after correction for multiple comparisons. In contrast, in controls, neural activation to angry faces was significantly greater than to happy faces, in the right inferior occipital (100–175 ms), the right calcarine (100–150 ms) and right postcentral (350–400 ms) gyri (Figure 4).

## Discussion

Our study highlights atypical neural activation to angry and happy faces in young adults with ASD using MEG. Despite comparable behavioral performance, young adults with ASD showed atypical recruitment of limbic and occipital regions occurring as early as 100 ms post-stimulus to both angry and happy faces. Between-group differences in neural activity emerged as early as 100–150 ms. In those with ASD, emotional faces elicited greater activation in traditional “emotion processing” areas including the anterior insula, fusiform gyrus, inferior temporal gyrus, ACC and amygdala. In contrast, controls showed greater activation, relative to adults with ASD, in occipital and parietal areas including the cuneus, precuneus, middle occipital gyrus, postcentral/supramarginal gyrus caudate, as well as the ACC. Between group differences in early occipital activity is consistent with a previous report of discrepant early visual processing to threat cues in youth with and without ASD (Malaia et al., 2017). Atypical recruitment of these regions in individuals with ASD may also indicate disparities in network dynamics (Malaia et al., 2016). In light of the lack of significant differences between angry and happy faces in individuals with ASD, in combination with similar patterns of between-group findings to both happy and angry faces, our results support a generalized atypicality in the neural mechanisms underlying affect processing in ASD.

### Anterior Insula Over-Activation in Young Adults with ASD

Implicated in a number of different functions such as salience (for a review see Uddin and Menon, 2009), attention (Eckert et al., 2009), interoception (Craig, 2003), empathy (Singer, 2006) as well as emotion processing (Silani et al., 2008), the right anterior insula is a paralimbic region with structural connectivity to areas such as the ACC (for a review see Bush et al., 2000) and amygdala (Mesulam and Mufson, 1982; Mufson and Mesulam, 1982), both key emotional processing areas, as well as to the nucleus accumbens, a structure that plays a key role in reward processing (Breiter et al., 2001; Craig, 2003; Menon and Levitin, 2005). In adolescents with ASD, early over-activation, relative to typically developing adolescents, in the right insula during implicit emotional face processing has been shown (Leung et al., 2015). Given that individuals with ASD lack interest in the human face (Osterling and Dawson, 1994) and do not derive social reward from positive faces (Sepeta et al., 2012), our data suggest that atypical activity in neural structures implicated in social reward contributes to the deficits in understanding social reward in ASD.

Additionally, the anterior insula is a key node in the salience network, which includes a number of brain regions that play a role in detecting, integrating and coordinating behavioral responses to relevant stimuli (Seeley et al., 2007; Menon and Uddin, 2010). In particular, the anterior insula has been implicated in engaging appropriate task-relevant networks involved in attention, working memory and higher cognitive processes while de-activating the default mode network (Sridharan et al., 2008). The differing activation to affective faces in individuals of this hub for the salience network, suggest that emotional faces do not have comparable salience in the ASD and control groups. This is congruent with findings of decreased accuracy in ASD compared to controls in the ability to detect happy and angry faces (Krysko and Rutherford, 2009, but see Ashwin et al., 2006). While hypoactivation in the anterior insula during social cognitive tasks is more likely to be found in ASD in fMRI studies (Di Martino et al., 2009), this meta-analysis included studies examining a broad range of social processes beyond affect processing (e.g., mentalizing). In addition, a more recent meta-analysis investigating emotional face processing in 13 studies using fMRI did not find either anterior insula over- or under-activation in individuals with ASD, compared to controls (Aoki et al., 2015). In light of these inconsistencies, involvement of the anterior insula during emotional face processing in ASD still warrants further investigation and highlights the fact that results may be affected by not only sample demographics or task demands but also neuroimaging modality. Furthermore, it is important to note that discrepancies between levels of activity in the anterior insula during social cognitive tasks do not counter findings of reduced connectivity between the anterior insula and other key social regions (Uddin and Menon, 2009; Leung et al., 2014). Nevertheless, atypical activity in the anterior insula in individuals with ASD indicates disparities in the ability to derive social reward from expressive faces, as well as the ability to engage networks that are subsequently involved in processing task-relevant or salient information. A direct link between increased neural connectivity and severity in social deficits in ASD has previously been established (Supekar et al., 2013).

### Greater Inferior Temporal and Fusiform Activity in Young Adults with ASD

We also found inferior temporal over-activation in response to both angry and happy faces in young adults with ASD compared to controls. Specifically, happy faces elicited elevated early (100–175 ms) left inferior temporal followed by right inferior temporal (300–425 ms) activity in individuals with ASD. Similarly, greater right inferior temporal (250–400 ms) activity in the ASD group was observed in response to angry faces. As inferior temporal activity is implicated in perception of facial identity (Haxby et al., 2000), these findings suggest that broad deficits in facial perception may weaken affect processing in individuals with ASD.

Previous findings in fMRI of reduced fusiform activity during face processing in ASD were modeled as a general face-processing deficit (Harms et al., 2010). Thus, our results of greater fusiform activity to both angry and happy faces in young adults with ASD in comparison to controls was unexpected, but consistent with atypical face processing in this population. Of particular interest was the laterality shift in fusiform activation following stimulus exposure; this pattern of early left followed by right fusiform over-activation in the ASD group compared to controls, was observed to both angry and happy faces, and may reflect multi-stage fusiform activation during face processing (Barbeau et al., 2008; Hung et al., 2010). This would also be concordant with increased featural processing, subsumed by the left fusiform, implying less automaticity in the ASD group.

### Atypical Amygdala and Anterior Cingulate Involvement during Affect Processing in ASD

Harms et al. (2010) have posited that compensatory mechanisms, or the recruitment of alternative methods of processing emotion, may signify atypical recruitment of neural regions implicated in pre-conscious stages of emotional processing. The amygdalae, known to facilitate rapid orientation towards threat stimuli (LeDoux, 1998) even when threat stimuli are masked, are an example of such structures (Breiter et al., 1996; Whalen et al., 1998). Our findings of elevated amygdala activity during emotional face processing in ASD to angry faces were not surprising given previous findings of elevated activity in those with ASD (Dalton et al., 2005; Monk et al., 2010). This may suggest a greater processing load on the amygdala while processing social stimuli in individuals with ASD.

The amygdalae and ACC have differing trajectories in developmental models of emotional processing, whereby, with age, subcortical systems involving the amygdalae mature earlier but are decreasingly engaged with age, while the frontal cortical regions, showing later maturation, may be increasingly engaged in emotion processing (Herba and Phillips, 2004). In support of this model, Hung et al. (2012) found a shifting involvement from the left to the right amygdala and an increase in the anterior cingulate activity with age. In the present study, angry, rather than fearful, faces elicited late right amygdala over-activity (400–450 ms) and simultaneous left anterior cingulate under-activity (400–500 ms) in adults with ASD. These results are consistent with our earlier findings of reduced ACC activation in adolescents with ASD (Leung et al., 2015). Collectively, these findings suggest that young adults with ASD recruit an immature and less efficient threat-processing system than controls.

### Happy and Angry Faces Elicit Distinct Neural Responses in Controls but Not in Young Adults with ASD

Angry, relative to happy, faces elicited greater right inferior occipital, calcarine and postcentral activity in controls. In contrast, there were no differential effects of emotion on neural activity in young adults with ASD. This lack of emotion-specific neural activity was also previously shown in youth with ASD (Malaia et al., 2017). This absence of neural sensitivity may underlie observations of atypical affective processing in individuals with ASD. Our findings are in line with those of Ashwin et al. (2007) who showed that, while neural responses differed according to varying intensities of fearful faces in typically developing adults, this effect was not observed in young adults with ASD. As the amygdala modulates the recruitment of visual areas involved in processing biologically relevant stimuli (Morris et al., 1998), the absence of a unique neural response to angry vs. happy faces in combination with atypical amygdala activity in our data suggest that the amygdalae are modulating other neural regions in an atypical manner in response to biologically relevant stimuli in ASD.

## Conclusion

This is the first study investigating emotional face processing in young adults with ASD using MEG. These novel findings differ from previous EEG and fMRI studies in yielding high spatial and temporal resolution regarding the atypical profile of neural activity during emotional face processing in young adults with ASD. Our results indicate that young adults with ASD show atypical recruitment of limbic and occipital regions occurring as early as 100 ms post-stimulus to both angry and happy faces, relative to their typically developing counterparts. These findings indicate that while young adults with ASD recruited brain areas traditionally implicated in affect processing, activity in these regions was atypical. Impairments in facial affect processing may be attributable to difficulty in deriving social reward from faces, ascribing salience to faces, and an immature threat processing system. Characterizing atypical patterns of neural activation will aid in understanding the maturation of atypical affective processing in ASD through young adulthood and ultimately, deficits in social cognition that are a hallmark of ASD.

## Author Contributions

RCL participated in task design, data collection, data analysis, results interpretation and manuscript writing. EWP participated in task design, data analysis, results interpretation and manuscript editing. EA participated in results interpretation and manuscript editing. MJT participated in task design, data collection, data analysis, results interpretation and manuscript editing.

## Conflict of Interest Statement

EA receives consultation fees (Roche and Takeda), royalties (Springer and APPI), unrestricted funding (Sanofi) and grant funding (SunapDx). The other authors declare that the research was conducted in the absence of any commercial or financial relationships that could be construed as a potential conflict of interest.

## Footnotes

^1^http://www.neurobs.com/

^2^http://www.fil.ion.ucl.ac.uk/spm/

^3^http://www.sccn.ucsd.edu/eeglab
